# Discriminating Malaria from Dengue Fever in Endemic Areas: Clinical and Biological Criteria, Prognostic Score and Utility of the C-Reactive Protein: A Retrospective Matched-Pair Study in French Guiana

**DOI:** 10.1371/journal.pntd.0002420

**Published:** 2013-09-12

**Authors:** Loïc Epelboin, Charlotte Boullé, Sihem Ouar-Epelboin, Matthieu Hanf, Philippe Dussart, Félix Djossou, Mathieu Nacher, Bernard Carme

**Affiliations:** 1 CIC-EC Antilles Guyane CIE 802 Inserm, Centre Hospitalier Andrée Rosemon, Cayenne, French Guiana; 2 Research team EPaT EA 3593, University of French West Indies and French Guiana, Cayenne, French Guiana; 3 AP-HP, Hôpital Pitié-Salpêtrière, Service de maladies Infectieuses et Tropicales, Paris, France; 4 UMI 233, Institut de Recherche pour le Développement (IRD)/Université Montpellier 1 (UM1), Montpellier; 5 French National Reference Centre for Arboviruses, Institut Pasteur de Guyane, Cayenne, French Guiana; 6 Infectious and Tropical Diseases Department, Centre Hospitalier Andrée Rosemon, Cayenne, French Guiana; Pediatric Dengue Vaccine Initiative, United States of America

## Abstract

**Background:**

Dengue and malaria are two major public health concerns in tropical settings. Although the pathogeneses of these two arthropod-borne diseases differ, their clinical and biological presentations are unspecific. During dengue epidemics, several hundred patients with fever and diffuse pain are weekly admitted at the emergency room. It is difficult to discriminate them from patients presenting malaria attacks. Furthermore, it may be impossible to provide a parasitological microscopic examination for all patients. This study aimed to establish a diagnostic algorithm for communities where dengue fever and malaria occur at some frequency in adults.

**Methodology/Principal Findings:**

A sub-study using the control groups of a case-control study in French Guiana – originally designed to compare dengue and malaria co-infected cases to single infected cases – was performed between 2004 and 2010. In brief, 208 patients with malaria matched to 208 patients with dengue fever were compared in the present study. A predictive score of malaria versus dengue was established using .632 bootstrap procedures. Multivariate analysis showed that male gender, age, tachycardia, anemia, thrombocytopenia, and CRP>5 mg/l were independently associated with malaria. The predictive score using those variables had an AUC of 0.86 (95%CI: 0.82–0.89), and the CRP was the preponderant predictive factor. The sensitivity and specificity of CRP>5 mg/L to discriminate malaria from dengue were of 0.995 (95%CI: 0.991–1) and 0.35 (95%CI 0.32–0.39), respectively.

**Conclusions/Significance:**

The clinical and biological score performed relatively well for discriminating cases of dengue versus malaria. Moreover, using only the CRP level turned to be a useful biomarker to discriminate feverish patients at low risk of malaria in an area where both infections exist. It would avoid more than 33% of unnecessary parasitological examinations with a very low risk of missing a malaria attack.

## Author Summary

The authors present a retrospective matched-pair study on dengue and malaria performed in French Guiana. These two infections are major public health concerns in tropical regions, especially in South America and Southeast Asia, where they affect neglected populations which makes them interesting to be published in a journal aiming to publish about neglected tropical diseases. Although the pathogeneses of these two arthropod-borne differ, their clinical and biological presentations are unspecific. During dengue epidemics, hundreds of patients are admitted weekly with diffuse pains and fever at the emergency room. Among them, it is difficult to accurately distinguish malaria attacks, which are far less frequent than dengue fever cases. Moreover, it may be impossible to provide a parasitological microscopic examination for all patients. We believe the results of the present study, based on a sample of n = 416 individual are worthwhile as they support evidence that biological factors can help to discriminate between the two, in areas where they co-exist in endemic areas. A simple prognostic score based on clinical and biological criteria was built, interesting and easy-to-use for physicians in tropical areas.

## Introduction

Dengue fever and malaria - the two most common arthropod-borne diseases - are major public health concerns in tropical settings [1], [2]. The dengue viruses (family *Flaviridae*, genus *Flavivirus*) and the *Plasmodium* parasites are widespread in American and Asian intertropical regions, where their endemic areas greatly overlap. Although their pathogeneses differ, their clinical and biological presentations are unspecific and it is difficult to distinguish the two infections.

**Figure 1:**
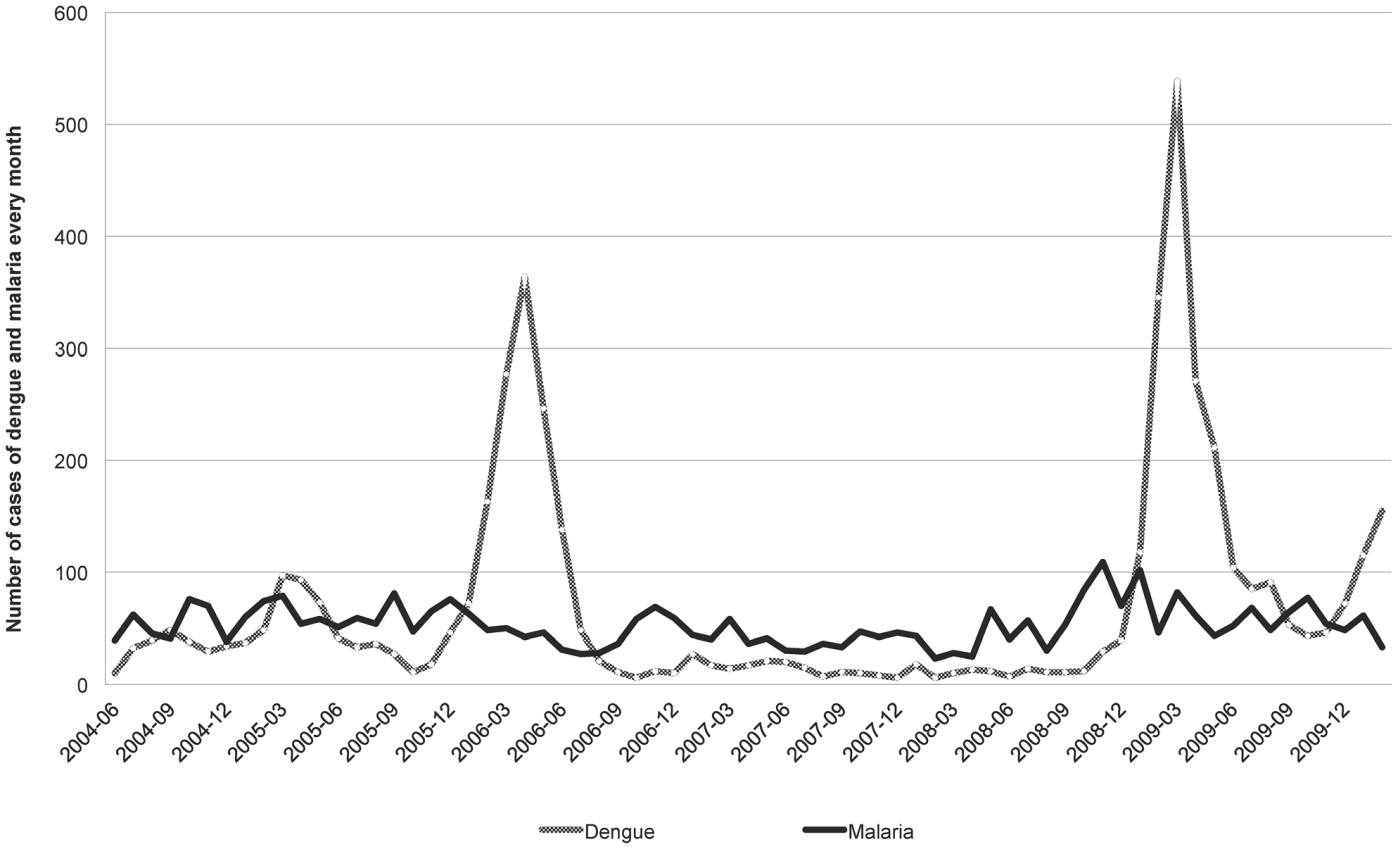
Monthly cases of dengue and malaria diagnosed in the Cayenne hospital between 2004 and 2010.

French Guiana is a French Overseas territory located on the northeastern coast of South America. About 90% of its 84,000 km^2^ surface is Amazonian rain forest. The remaining 10% in the north is a coastal plain where 90% of the 215,000 inhabitants live. Cayenne is the capital and represented almost half of the population in 2009 [3]. In French Guiana, dengue fever (DF) and malaria coexist, and are both frequent. Malaria is endemic with an annual number of cases ranging from 3,200 to 4,700 [4] (Figure 1). Until 2006, *P. vivax* represented half of the total cases, but currently, the proportion of *P. vivax* malaria is 75%, similarly to other observations in Latin America [2], [4], [5]. Dengue fever is endemo-epidemic and the 4 serotypes (DENV-1, DENV-2, DENV-3 and DENV-4) circulate. If the first cases of DF were reported in French Guiana in 1943, an increase in the number of DF cases and outbreaks has been observed recently [6]. The two last main epidemics occurred in 2006 and 2009, reaching a total of 14.000 estimated cases. During dengue epidemics, hundreds of consultations are held in the emergency department, and it is a real challenge for physicians to identify patients with a high risk of malaria among all those with dengue fever. Demographic criteria - that have never been evaluated - are commonly used to target those patients who should get a parasitological examination.

The main objective of the study was to identify clinical and biological criteria to discriminate malaria from dengue fever in endemic areas. The secondary objective was to build a predictive score of malaria to guide the indication of parasitological examinations in case of a dengue epidemic. Thus, this study aimed to establish a diagnostic algorithm for communities where dengue fever and malaria occur at some frequency in adults.

## Methods

### Study population

A sub-study of a matched retrospective case-control study – designed to compare dengue and malaria co-infections to single infections – was performed between June 2004 and February 2010 at the Emergency Room (ER) of Cayenne Hospital in French Guiana [7]. In brief, the patients co-infected with dengue and malaria were exhaustively identified during the study period (n = 104) and matched to 4 controls −2 with malaria only and 2 with dengue only – based on the date of the microbiological diagnosis. For the present study, we included the two groups of n = 208 mono-infected patients to compare those with malaria to those with dengue fever.

### Case definitions

Case definitions were based on compatible clinical history and positive biological diagnosis for one infection and negative for the other.

The diagnosis of malaria relied on the identification of hematozoa on a thin blood film and/or on a thick blood film stained with Giemsa and a negative dengue test. During the study period, the diagnosis method for malaria diagnosis (microscopic examination by an expert microscopist) and parasitaemia evaluation remained unchanged. Thick and thin blood films were prepared within an hour of blood collection (4 ml of venous blood collected in an EDTA tube). They were stained with diluted Giemsa (1∶10). Two hundred oil immersion fields of the thin blood film and 1000 white blood cells (WBC) on the thick smear were examined before classifying a slide as negative. The screening sensitivity was ∼6 plasmodia/µl (assuming 8,000 WBC/µl of blood). At the time of the study period, malaria rapid diagnosis tests (RDT) were not routinely performed.

Due to the evolution of the techniques between 2004 and 2010, the laboratory diagnosis of dengue relied on different methods. Before 2006, the diagnosis was based on direct virus isolation and genome detection by RT-PCR (Reverse Transcriptase-Polymerase Chain Reaction). From 2006, the hospital protocol was modified in accordance with evidence presented by Dussart et al. [8], [9] and confirmed by a multinational prospective clinical study carried in South-East Asia and Latin America, in which the protocol was found to have good sensitivity [10]. NS1 antigen detection was thus performed from day 0 to day 5, and indirect diagnosis based on detection of specific anti-dengue IgM antibodies in patients' sera was performed after day 3 [11]. On days 4 and 5, both tests were made and associated to dengue RT-PCR. Eventually, 56 (26.9%) patients were diagnosed using cell-culture virus isolation and/or RT-PCR, 84 (40.4%) using NS1 antigen +/− IgM, 58 (27.8%) using IgM, 9 using IgM+IgA, and one had IgM seroconversion. The proportion of dengue hemorrhagic fever (DHF) among all DF was not assessed, given the difficulties to establish the diagnosis of DHF retrospectively following the WHO 1997 definition (positive tourniquet test, rising hematocrit, and elevated thrombocytopenia) [12].

### Data collection

Individual data (including socio-epidemiologic data, previous medical history, clinical symptoms, and biological results) were retrieved anonymously from the computerized medical charts. Data were analyzed using R version 2.13.0, the Epicalc package, and Stata 11.0 software (Stata Corporation, College Station, USA).

### Statistical methods

Continuous variables were categorized following laboratory or usual cut-off values. Because of the small sample size, they were generally dichotomized in order to preserve statistical power. A C-reactive protein (CRP) level ≤5 mg/L was considered as negative. Continuous variables were compared using matched Student t-test. Categorical variables were analyzed using the Wald test in matched bivariate analysis. Statistical significance was set at p<0.05. Because of the unreliability of the data obtained retrospectively, variables obtained from anamnesis and clinical examination, and variables with >5% missing data were *a priori* excluded from the multivariate model. Remaining variables with p<0.20 in bivariate analyses were entered into a conditional multivariate logistic regression model. Conditional multivariate backward stepwise logistic regression was then performed to estimate the adjusted odds ratios (OR) and 95% confidence intervals (95%CI).

### Predictive score

All significant variables of the logistic model were used to determine a preliminary predictive score of malaria versus dengue. To improve model stability, a semi-external validation was performed using a .632 bootstrap procedure [13]. Briefly, 1000 training and testing samples were generated. Medians and interquartile ranges of each β coefficient obtained from regression over the training samples were then reported. To assess the goodness-of-fit model, the AUC (area under the receiver operating characteristic curve) and its 95%CI were computed. Median β coefficients from the bootstrap procedure were then rounded to construct integer weights for each variable. The assigned weights were then summed to compute that patient's score. The intrinsic qualities of the score (sensibility and specificity) were then assessed using a classic bootstrap procedure. All analyses were performed using R software, version 2.13.0, and Stata 11.0 software (StataCorp).

### Ethics statement

The retrospective use of anonymous patient files on the site of patient care is authorized by the French National Commission on Informatics and Liberties. All the data collected retrospectively were anonymized in a standardized case report form and in the database.

## Results

### General characteristics of the study population (Table 1)

**Table 1:** Comparison between malaria and dengue epidemiological and clinical variables by matched bivariate analysis.

	*DENGUE (n = 208)* *N (%)*	*MALARIA (n = 208)* *N (%)*	OR*	95% CI*	p**
**Male gender**	119 (57,2)	162 (77,9)	2,65	1,71–4,11	**<0,001**
**Age <15 years old**	49 (23,6)	18 (8,7)	0,31	0,18–0,56	**<0,001**
**Journey in forest**	7 (3,4)	51 (24,6)	10,27	4,3–24,49	**<0,001**
**Inhabitant of the coast** ¥	183 (92,9)	151 (74,4)	0,27	0,16–0,45	**<0,001**
**Hospital stay ≥2 days**	71 (34,1)	72 (34,6)	1,03	0,69–1,54	0,89
**Admission**	45 (21,6)	69 (33,2)	1,78	1,16–2,75	**0,009**
**Medical history of malaria**	21 (10,2)	115 (62,8)	16,15	8,3–31,45	**<0,001**
**Duration of fever >5 days**	33 (16,3)	61 (29,9)	2,20	1,36–3,58	**0,001**
**Tachycardia (>90 bpm)**	128 (64,6)	165 (82,1)	2,43	1,52–3,89	**<0,001**
**Low blood pressure (<90 mmHg)**	3 (1,8)	7 (3,6)	1,81	0,46–7,11	0,39
**Fever ≥40°C**	10 (4,9)	24 (11,9)	2,64	1,21–5,74	**0,01**
**Headaches**	122 (59,2)	126 (63,3)	1,18	0,8–1,76	0,40
**Retro-orbital pains**	26 (12,6)	10 (5)	0,37	0,17–0,8	**0,01**
**Asthenia**	53 (25,9)	56 (28,3)	1,11	0,73–1,7	0,62
**Myalgias**	102 (49,8)	109 (54,5)	1,19	0,81–1,76	0,37
**Chills**	37 (18)	64 (32,2)	2,16	1,34–3,48	**0,002**
**Rash**	30 (14,6)	5 (2,5)	0,16	0,06–0,42	**<0,001**
**Digestive symptoms**	127 (62)	130 (64)	1,07	0,7–1,62	0,76
**Malaise et/ou dizziness**	16 (7,8)	7 (3,4)	0,38	0,15–0,97	**0,04**
**Splenomegaly**	3 (3,1)	23 (18,3)	19,21	2,48–148,78	**0,005**
**Jaundice**	6 (2,9)	9 (4,3)	1,53	0,53–4,4	0,43
**ENT symptoms** §	42 (20,2)	10 (4,8)	0,21	0,1–0,42	**<0,001**

* *Odds ratio* (OR) and 95% confidence interval (95% CI) and p calculated by matched bivariate analysis.

** p-value calculated with Wald test in matched bivariate analysis.

¥ Inhabitants of the coast are defined as people living in Cayenne, Rémire-Montjoly, Matoury Macouria, Kourou, Irakoubo or Mana.; Other people declared to live in Cacao, Roura, Montsinnéry-Tonnégrande, Régina, Saül, St-Elie, Saint Laurent du Maroni, Apatou, Maripasoula, Papaïchton, St George de l'Oyapock, Camopi or Trois-Sauts. 4 people came from France, 1 from the French Caribbean and 1 from French Polynesia.

Bpm, beats per minut;

§ ENT symptoms, (Ear, Nose and Throat): pharyngitis, otitis and/or sinusitis).

The 416 patients were mostly men (67.6%, sex ratio = 2) and had a median age of 30.7 years (interquartile range (IQR): 20.6–42.3; range: 0.4–86 years). Sixty-seven patients (16.1%) were younger than 15.

### Microbiological results

Among the 208 malaria patients, 141 (68%) were infected with *P. vivax*, 58 (28%) with *P. falciparum*, and 8 (4%) with both species.

In the DF group, 140 (67%) cases were early diagnosis (NS1 antigen, RT-PCR or IgM seroconversion) and 68 (33%) were delayed diagnosis (IgM). The results of serotyping were available for 91 patients, among whom 25 were infected by the DENV-1 (27.5%), 25 by the DENV-2 (27.5%), 28 by the DENV-3 (30.8%), and 13 by the DENV-4 (14.2%).

### Comparison between malaria and dengue fever in bivariate analysis (Tables 1 & 2)

**Table 2:** Comparison between malaria and dengue biological variables by matched bivariate analysis.

	*DENGUE (n = 208)* *N (%)*	*MALARIA (n = 208)* *N (%)*	OR*	95% CI*	p**
**Anemia (Hemoglobin <12 g/dl)**	40 (19,2)	43 (20,7)	1.13	0.69–1.84	0.63
**Low Hematocrit (<36%)**	39 (18.8)	52 (25.9)	1,50	0,93–2,42	0,10
**Leukocytosis (>10×10^9^/L)**	13 (6.3)	2 (0.1)	0,15	0,03–0,66	**0,01**
**Lymphopenia (<0.5×10^9^/L)**	57 (27,4)	48 (23,1)	0,80	0,52–1,24	0,32
**Thrombocytopenia (<100×10^9^/L)**	25 (12.0)	102 (49.0)	7,45	4,32–12,83	**<0,001**
**PT<70%**	19 (13,9)	22 (15,4)	0,85	0,39–1,86	0,69
**ATT>1,2**	19 (14,1)	12 (8,5)	0,34	0,12–0,96	**0,04**
**Urea>8 mmol/L**	3 (1,5)	14 (7)	5,21	1,46–18,53	**0,01**
**Creatinin >111 µmol/L**	4 (2)	14 (7)	3,66	1,2–11,16	**0,02**
**Bilirubin >50 µmol/L**	3 (1,6)	20 (10,5)	7,02	2,02–24,43	**0,002**
**ALT>2N**	32 (16)	33 (16,7)	0,99	0,58–1,68	0,96
**AST>2N**	41 (20,5)	23 (11,6)	0,49	0,28–0,86	**0,01**
**CRP<5**	73 (35,3)	1 (0,5)	-	-	**<0,001**
**CRP 5 à 50**	115 (55,6)	78 (38)	39,79	5,44–291,12	**-**
**CRP>50**	19 (9,2)	126 (61,5)	306,84	40,22–2340,61	**-**

* *Odds ratio* (OR) and 95% confidence interval (95% CI) and p calculated by matched bivariate analysis.

** p-value calculated with Wald test in matched bivariate analysis.

ALT, alanine aminotransferase; AST, aspartate aminotransferase; CRP, C-reactive protein.

Patients with malaria were significantly older than those with dengue (35.3 years (±15.4) vs. 28.0 years (±17.85) respectively; p<0.001). Variables significantly associated with malaria were age ≥15 years, male gender, recent journey in the forest, and previous history of malaria. Residency on the coast was associated with dengue.

Eventually, the clinical outcomes were favorable in both groups, as no deaths were reported. The malaria patients arrived in the ER later than those with dengue (duration of the symptoms 5.1 days (±3.9) vs. 3.7 days (±2.4), respectively; p<0.001) and were more often hospitalized.

Tachycardia >90 bpm, fever ≥40°C, chills, malaise and/or dizziness, and splenomegaly were more frequent in malaria patients. Conversely, retro-orbital pain, ENT symptoms (such as associated pharyngitis, otitis or sinusitis), and rash were associated with DF. However, there were no differences between the two groups in terms of blood pressure, signs of shock, fatigue, headaches, arthromyalgia, digestive disorders, dehydration, pallor, or jaundice.

The mean platelet count was higher in patients with DF (198.10^9^/L (±93)) than in those with malaria (110.10^9^/L (±55)) respectively; p<0.001).

Thrombocytopenia <100.10^9^/L, kidney failure, electrolyte abnormalities (eg, hyponatremia, hypokaliemia,and acidosis) and hyperbilirubinemia were significantly more frequent in malaria patients than in those with DF. An elevated CRP (>5 mg/L) was significantly associated with malaria (p<0.001). The proportion of patients with CRP>50 mg/L was 69.0% in *P. falciparum* infections, 58.7% in *P. vivax* infections, and 65.5% in patients infected with both species. There was no significant difference between the Plasmodia association and *P. falciparum* alone nor or between *P. vivax* and *P. falciparum* (p = 0.420 and 0.170 respectively with Fisher's exact test) for the proportion of high CRP level.

Biological variables significantly associated with dengue fever were prolonged activated partial thromboplastin time (aPTT), and elevated ALT levels. Finally, anemia, neutropenia, or lymphopenia did not differ between dengue and malaria patients.

### Risk factors and prognostic score of malaria vs. dengue fever

In matched multivariate analysis, male gender, age>15 years, tachycardia, anemia, thrombocytopenia and CRP>5 mg/l remained independently associated with malaria as compared to DF (Table 3).

**Table 3:** Median β coefficients estimated by multivariate logistic regression model and bootstrapping procedure.

	Matched multivariate analysis			.632 bootstrap	
	OR*	95% CI*	p*	β-coefficients	Value of score
**Heart beat >90 bpm**	2,50	1,35–4,63	0,004	0,915	1
**C-reactive protein >5 mg/l**	88,12	10,16-764-45	<0,001	9,322	9
**Age >15 years old**	2,99	1,32–6,75	0,008	1,235	1
**Platelet count <100.10^9^/L**	10,33	2,10–50,74	0,004	2,185	2
**Hematocrite <36%**	2,12	1,05–4,29	0,037	1,044	1
**Male gender**	2,92	1,64–5,19	<0,001	1,132	1

* *Odds ratio* and 95% confidence interval and p-value calculated by matched multivariate analysis.

Matched multivariate logistic regression analysis was performed for malaria and dengue. Weights are based on the β coefficients of the logistic regression and were calculated by rounding the model coefficients to the nearest whole integer after .*632* bootstrapping (median). The weights rank the risk predictors in relative importance and dictate how one assigns integer points value for each predictor for a given patient. The assigned points are then summed to compute that individual's risk for the mixed clinical and biological malaria score values.

**Figure 2:**
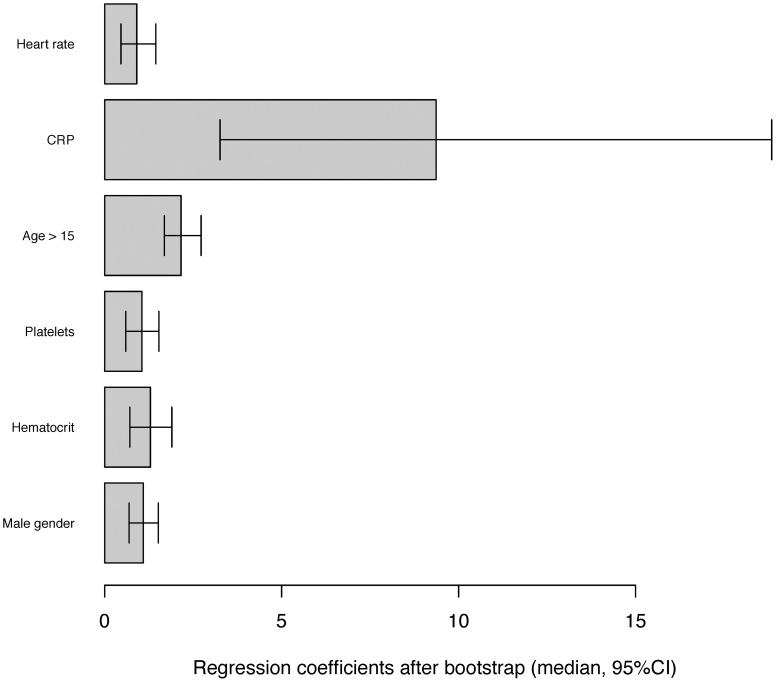
Representation of the beta coefficients from bootstrap resampling of clinical and biological variables.

**Figure 3:**
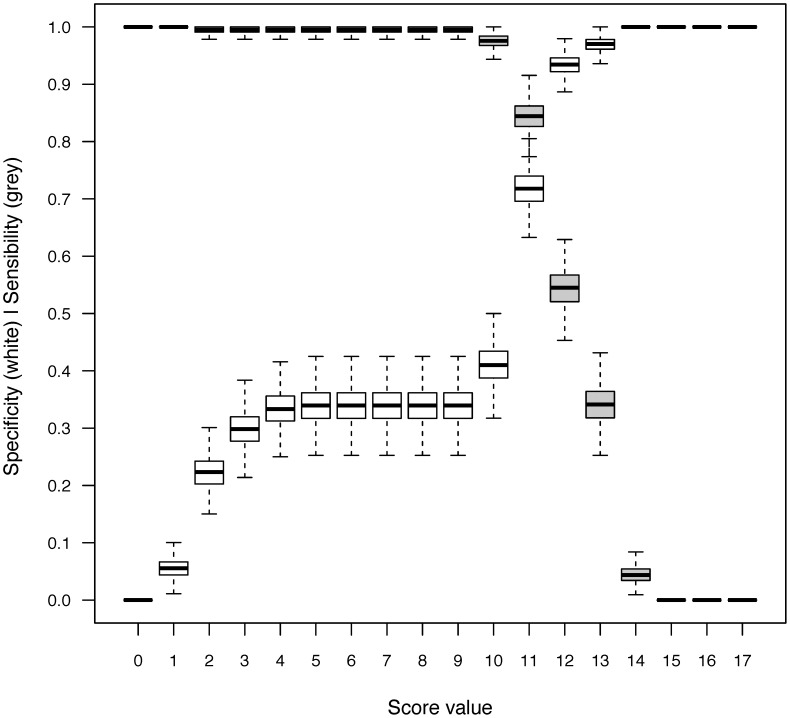
Sensitivity and specificity curves of the mixed clinical and biological malaria vs. dengue predictive score.

After 1000 resampling using a .632 bootstrap procedure, the median β coefficients matching these prognostic factors were estimated. The median AUC was 0.86 (95% CI, 0.82; 0.89), which indicates good model discrimination. The construction of the score is detailed in Table 3. The median β coefficient of each prognostic factor was rounded to a weight of +1, except for platelet count (+2) and CRP level (+9) (Figure 2). For each patient, the score was then obtained by summing the corresponding weights, and ranged from 0 to 15. The sensitivity and specificity of the different cut-offs are presented in figure 3. Placing the highest cost on failing to diagnose malaria, the cut-off of 10 was chosen to increase the sensitivity and design a screening test. Patients with a score <10 were at very low risk of malaria (Figure 3), with a sensitivity of 0.997 (95% CI, 0.995–1) and a specificity of 0.41 (95% CI, 0.32–0.50). Negative and predictive values could not be estimated because the sample size had been pre-determined.

Considering the large weight of the CRP>5 mg/L, sensitivity and specificity for this coefficient alone were estimated (Table 4). The sensitivity and specificity of CRP alone was of 0.995 (95%CI 0.991–1) and 0.35 (95%CI 0.32–0.39), respectively.

**Table 4:** Repartition of the dengue and malaria patients depending on the C-reactive protein (CRP) level.

	CRP ≤5 mg/L	CRP>5 mg/L	Total
**Malaria**	1	204	205
**Dengue fever**	73	134	207

## Discussion

In the present study, the clinical presentation was more severe and biological abnormalities were more frequent in patients with malaria than in those with dengue fever, despite that DF and DHF were both included and pooled in the study. However this might be partly due to the fact that patients with dengue presented early after the onset of symptoms, when thrombocytopenia and DHF features generally occur later in the evolution of the disease.

If it is usually assumed that *P. falciparum* is the most likely *Plasmodium* specie to cause severe malaria, there is growing evidence in the literature that *P. vivax* can also cause severe complications [14]. Furthermore, clinical features could not differentiate the infecting *Plasmodium* species in Karen children living in another area of low transmission, in Thailand [15]. For this reason, and also because we aimed at determining the risk factors for patients requiring anti-malarial treatment, the infections by both species were pooled indifferently in the present study. The association of retro-orbital pain, ENT symptoms, and skin rash with dengue that we reported is also consistent with what is found in the literature [16]. The proportion of patients with DHF was not assessed given the difficulty to retrospectively establish this diagnosis using the 1997 WHO definition (positive tourniquet test and rising hematocrit) [12].

A low platelet count is classical in both infections. In many tropical regions, fever with thrombocytopenia is assumed to be malaria attack, and fever with thrombocytopenia and negative blood smear is considered to be dengue fever [17]. In the present study, thrombocytopenia (<100.10^9^/L) was more frequent in patients with malaria than in those with dengue. A study comparing the biological features of dengue, malaria and leptospirosis in India showed similar results, observing that thrombocytopenia was a predictor of malaria [18].

The CRP is a protein found in the blood that is synthesized by the liver. It is mainly used as an inflammation biomarker, and particularly for bacterial infections. We found that an elevated CRP level was independently associated with malaria. Few reports have been published about the values of CRP in malaria patients, and generally showed that it is elevated, as reported in a Zambian study [19]. A study on 25 patients with imported *P. falciparum* malaria from Africa showed that the CRP had a better positive predictive value than the erythrocyte sedimentation rate [20]. In a study performed among patients with *P. falciparum* malaria imported in Sweden, only 1 out of 58 patients had a normal CRP [21]. Nevertheless, in this study, the CRP did not allow to differentiate malaria from other causes of fever in travelers. Moreover, CRP has already been used as a biomarker of malaria in febrile patients returning from tropical areas and in epidemiological investigations in endemic areas [22]. Similarly, a CRP level <50 mg/L helped to differentiate dengue fever from leptospirosis in New Caledonia [23].

Previous studies already aimed to establish criteria to differentiate malaria attacks from other endemic infections such as dengue fever. A study performed in southern India compared malaria to other infectious causes of fever (including dengue), and the arguments for malaria attack were normal WBC, moderate to severe thrombocytopenia, renal failure, splenomegaly, hyperbilirubinemia and cytolysis; meanwhile criteria for DF were having a rash, hemorrhagic manifestations, normal to low WBC, moderate to severe thrombocytopenia, and hypertransaminasemia [24]. A pediatric Indian study conducted on children with malaria, dengue or leptospirosis found that splenomegaly and a lower platelet count were more frequent in malaria patients than those with dengue. Elevated ALT and hepatomegaly were seen in dengue [18].

During dengue epidemics, more than 500 people may consult weekly at the Cayenne ER for febrile syndrome, resulting in up to 130 biologically confirmed dengue and 5 to 25 malaria cases (Figure 1). The ER and the laboratory facilities are often overwhelmed during these epidemics, and it is difficult to achieve a parasitological examination for all febrile syndromes, as it requires time, human resources and experienced laboratory technicians.

In French Guiana, dengue is classically found on the coast and settled in urban areas [25], where most people live, while malaria is more frequently contracted in the Amazonian forest and along the two bordering rivers (Oyapock and Maroni) [5], [26]. For these reasons, emergency physicians empirically do not ask for parasitological examination for the patients who do not report a stay in these locations even if the general recommendations are to systematically perform the test in patients with fever living or returning from endemic areas [27]. The present study confirms that socio-demographic characteristics differ between dengue and malaria patients. Schematically, patients with malaria attack were adult males reporting at least one journey in the forest (military, woodworkers, gold-miners, or forest entertainment) while patients with dengue were generally younger (a quarter being under 15 years-old,) with a more balanced gender distribution, lived on the coast and did not report going into the rain forest.

To our knowledge, if criteria were investigated to differentiate malaria or dengue fever from other diseases, no predictive score had been established (figure 2). The aim of the present study was not to build a score to challenge the indication of the parasitological examination. It meant to guide the prescription of thick smears, in the ER located in areas endemic for both dengue and malaria, especially during DF epidemics when dengue cases are 25 fold more frequent than malaria cases (125 vs. 5) (Figure 1).

Here this therapeutic attitude could be proposed with a score ≥+10, which corresponds to a CRP level greater than 5 mg/L associated to any one criteria among: male gender, age>15 years, tachycardia, anemia, or thrombocytopenia.

Considering the score results, using the CRP level alone is more useful on several levels. First, tachycardia, age, thrombocytopenia and hematocrit have a very low weight (1 to 2) in the score compared to the CRP level that is affected by a weight of +9, which is very close to the cut-off. Furthermore, the complete score uses parameters that are quite specific of the local Guianese epidemiology (age, gender). In this regard, using only the CRP level would allow better extrapolation of this score to other areas. The sensitivity of the CRP does not significantly differ from the previous score, but its specificity is lower. Nevertheless, the aim of the present study was to find a way to avoid a large number of unnecessary parasitological examinations without failing to diagnose malaria attacks. Using the CRP level with a cut-off inferior to 5 mg/L to challenge the indication of the thick smear induced a reduction of 35% of the unnecessary parasitological tests, and led to only one misdiagnosis of malaria attack out of 205. The rising availability of rapid tests for dengue and malaria may diminish the interest of CRP to differentiate these two major diseases. Nevertheless, the use of the CRP, and maybe, the use of CRP RDT, can also help to differentiate dengue from bacterial infection. Thus, a study was published in a pediatric cohort, showing the great interest, in terms of avoiding useless tests, of using CRP RDT (microCRP) for the diagnosis of bacterial infection in children [28], [29]. Therefore, from a broader perspective, implementing such a rapid diagnostic test could be interesting to differentiate malaria and any bacterial infection from dengue in endemic areas, especially during epidemics. Finally, in case of positive dengue RDT, an elevated CRP level may hint at looking for malaria or bacterial co-infection [7]. In our study, more than 50% of DF patients with CRP>50 mg/L had an associated bacterial infection, such as otitis, sinusitis or urinary tract infection.

Finally, the CRP level was a useful, sensitive and simple tool to identify patients at low risk of malaria during DF epidemics and to guide the decision of parasitological examination, without replacing the thick smear. Nevertheless, an external validation is necessary in further prospective studies, as is the comparison of cost-effectiveness of the CRP level vs. the RDT which are widely available worldwide but were not used in the present study.

### Conclusion

In many tropical areas malaria and DF coexist. Due to the challenge posed by their similar clinical pictures in case of massive influx of patients with “dengue-like” syndrome during an epidemic of DF, a predictive score was built to target the patients with a high risk of malaria and who needed parasitological examination.

To our knowledge the present study is the first to propose a simple and sensitive tool, the CRP level, to discriminate malaria from dengue fever. If it does not replace the microscopic examination, it helps to avoid one third of the unnecessary parasitological tests for patients at very low risk of malaria. In case of documented dengue, an elevated CRP level may lead to seek an associated diagnosis such as malaria or bacterial infection.

## Supporting Information

Checklist S1 STROBE checklist.
(DOC)
